# Pleomorphic adenoma of a subconjunctival ectopic lacrimal gland

**DOI:** 10.4103/0301-4738.62656

**Published:** 2010

**Authors:** S Patyal, Ajay Banarji, Madhu Bhadauria, V S Gurunadh

**Affiliations:** Department of Ophthalmology, AFMC, Sholapur Road, Pune – 40, India

**Keywords:** Ectopic, lacrimal gland, pleomorphic adenoma

## Abstract

This report describes a case of pleomorphic adenoma of an ectopic lacrimal gland arising subconjunctivally in the lateral fornix in a 13-year-old girl. The tumor was removed surgically in toto with the capsule. This is probably the first reported case.

Ectopic lacrimal tissue has been found in the orbit, lids and adnexa and also within the globe. Ninety-one cases of ectopic lacrimal tissue have been reported in literature, of which 14 have been found in the globe. This ectopic lacrimal tissue itself may pose to be a diagnostic dilemma. Neoplastic transformation of such tissue may occur. Pleomorphic adenoma is a common tumor seen in the lacrimal gland and has been found rarely to occur in accessory lacrimal glands. It has not yet been reported in an ectopic lacrimal gland in a younger age group.

## Case Report

**Figure 1:**
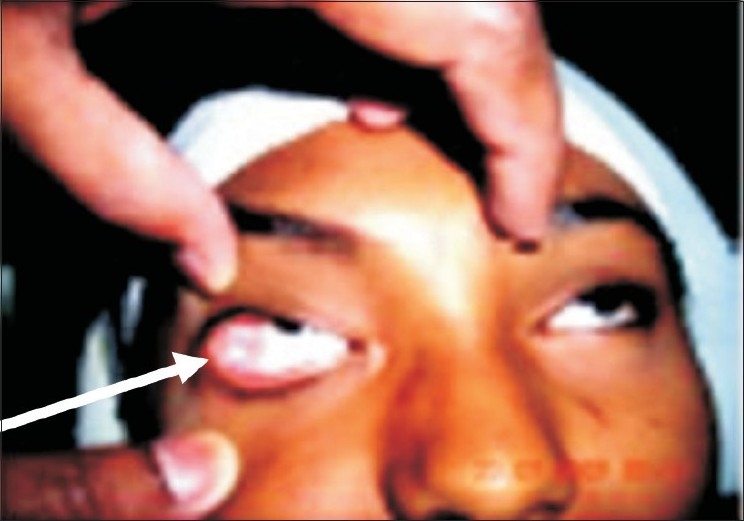
Pre operative photograph of the lesion protruding from the lateral canthus of right eye (arrow pointing at tumour).The upper lid has been pulled up. Part of everted tarsal plate present

A 13-year-old female patient reported in June 2007 with complaints of a slowly growing painless swelling in the lateral fornix of the left eye of six months duration [Fig. 1]. There was no history of trauma, pain, and lacrimation, restriction of ocular movements or impairment of vision. No other swelling was present elsewhere in the body.

Systemic examination showed no abnormality. Examination of the left eye revealed a firm, nodular, nontender, nonpulsatile, irreducible swelling in the lateral canthus. The conjunctiva over the swelling was freely movable. On adduction the swelling indented the eyeball. There was mild conjunctival congestion over the swelling. Ocular examination was otherwise within normal limits with visual acuity of 20/20. Ocular movements were full and free. Right eye examination was within normal limits.

Relevant investigations consisting of hemogram, total and differential counts (done to exclude infective etiology) were within normal limits. Non-contrast-enhanced computerized tomography scan of orbits revealed a homogenous soft tissue density mass in the lateral canthus of the left eye not infiltrating the surrounding tissues, suggestive of a benign tumor. Ultrasonography showed medium to high reflectivity with moderate sound attenuation with well-defined contour suggestive of a dermoid.

**Figure 2:**
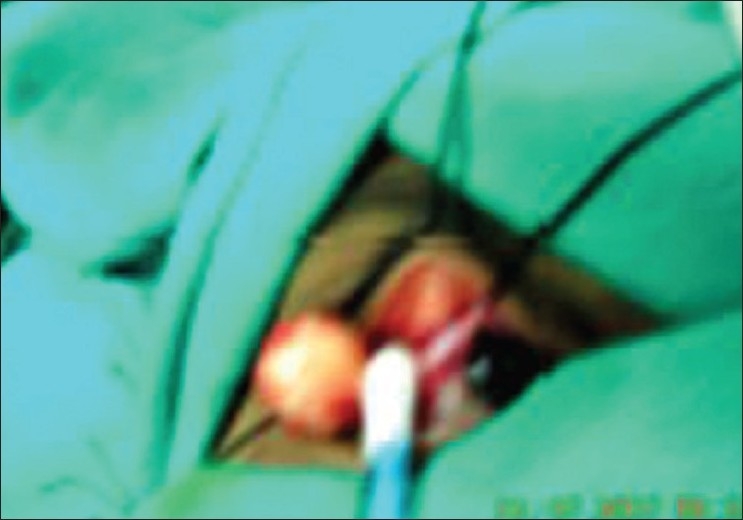
Per operative photograph of the entire tumour. Tumour removed completely after dissecting it from Tenon's capsule and conjunctiva

**Figure 3:**
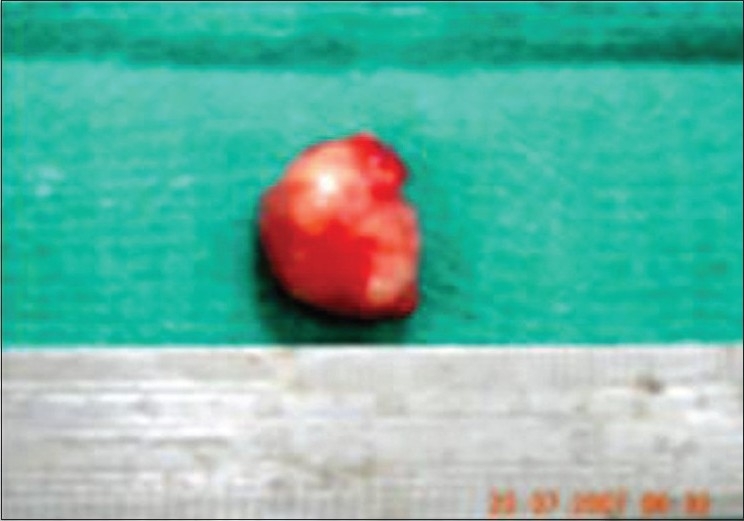
Tumour dimensions measured by a scale 2×1.5×1cms

The lesion was explored by a vertical incision in the conjunctiva overlying the swelling. A similar incision was made on the Tenon's capsule and a firm slightly lobulated swelling, free from other structures was found deep in the lateral fornix which was removed in toto by blunt dissection with an intact capsule [Fig. 2]. This tumor had no connection with any other structure in the orbit. The tumor measured 2×1.5×1cm [Fig. 3]. The Tenon's capsule and conjunctiva were closed in layers.

**Figure 4:**
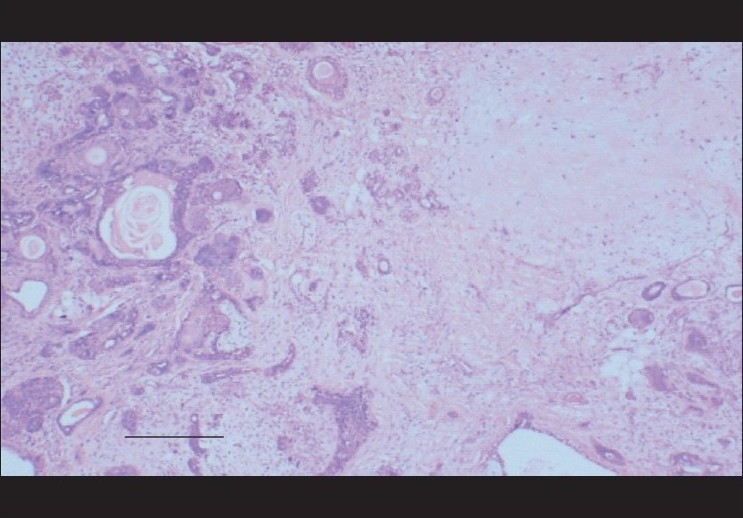
Histopathology of Pleomorphic adenoma: Sections show a cellular lesion with biphasic pattern. There is epithelial admixture. The epithelial elements are in glandular profile with myoepithelial proliferation. Foci of squamous metaplasia are seen. Stroma is chondromyxoid in nature. No atypia is noted. Immunochemistry: Ductal epithelial component is positive for keratin (CK 19), EMA and CEA. Myoepithelial component is positive for keratin, actin SMA and S- 100.OPINION – Pleomorphic Adenoma Magnification – 10×

The postoperative period was uneventful. Histopathology revealed the tissue to be a pleomorphic adenoma showing a biphasic cellular nature. The tumor tissue comprised epithelial elements arranged as trabeculae, glands and cysts. Foci of squamous metaplasia were present. The epitheloid cells were cuboidal to columnar. The stroma was chondromyxoid. No malignant component was present [Fig. 4]. The stain used was hematoxylin-eosin. The patient has been observed for more than one year with no recurrence of the swelling.

## Discussion

Lacrimal gland tissue is present in the orbital and palpebral lobes of the lacrimal gland, in the accessory glands of Krause and Wolfring.[1] Ectopic lacrimal gland tissue may be found in the caruncle, bulbar conjunctiva, outer canthus, and lower lid, intraorbital and intraocular regions and in any location in and around the eye.[2] In this patient, ectopic lacrimal gland was present beneath the bulbar conjunctiva.

The lacrimal gland develops from basal conjunctival cells as solid buds at eight weeks gestation.[3] These buds gradually migrate and finally come to lie in the lacrimal gland fossa. The tissue continues to grow after birth and complete differentiation occurs only after three years of age. The accessory lacrimal glands are similarly formed as ectodermal evaginations of the conjunctiva. During this process of development a part of the gland may get sequestered and develop separately, unconnected to the main mass of the lacrimal gland.[4] Various other theories for the development of ectopic lacrimal tissue have also been propounded – implantation of lacrimal tissue with surface epithelium during lens formation, lacrimal gland buds being pinched off during closure of the choroidal fissure especially when they are in close proximity and intraocular extension of lacrimal tissue along preexisting scleral defects which may close later. There can also be epithelial- mesenchymal interaction in which FGF-10, inductive signal intensity for the lacrimal gland acts directly on the conjunctival epithelium stimulating it to proliferate.[5]

Pleomorphic adenomas in the orbit can be found in the lacrimal gland,[6] palpebral lobe of the lacrimal gland associated with raised intraocular pressure,[7] in the upper lid,[8] in the lower lid developing in Krause's gland,[9] lacrimal sac and orbit. The current tumor had no connection with the lacrimal gland and was situated deep in the subconjunctival tissue in the lateral fornix; it most likely was a tumor arising from ectopic lacrimal tissue. The palpebral portion of the lacrimal gland anatomically is so placed that its anterior border lies just above the outer part of the upper fornix and can be seen in this situation through the conjunctiva when the upper lid is everted (Wolff's Anatomy). The current tumor was present in the lateral fornix as shown by an arrow in Fig. a. Unusual presentations of pleomorphic adenomas have also been reported in the literature. Christie *et al.* (1995) excised a well-circumscribed mass from the lacrimal gland fossa of the right orbit of a 57-year-old woman which was found to be a combination of a small benign mixed tumor and a large ductal cyst of the lacrimal gland.[10] A giant pleomorphic adenoma of the lacrimal gland that concealed a blind eye for six years in a 24-year-old, seen protruding from the superolateral left upper lid was excised by Guerra *et al.*[11]

In this patient, pleomorphic adenoma developed in ectopic lacrimal gland tissue at the age of 13 years whereas it is usually found between the 30-70 years. Cases of pleomorphic adenoma have also been found between 6-80 years of age. This is an unusual and rare presentation of pleomorphic adenoma in an ectopic lacrimal gland tissue in a young female, presenting subconjunctivally. Literature was searched through search engines Pubmed, Google and Yahoo in all languages, with key words ectopic lacrimal gland for which references were obtained. These stated that ectopic lacrimal tissue can occur in lids, caruncle, bulbar conjunctiva, outer canthus, intraocular and intraorbital regions proving this tumor originate from ectopic lacrimal tissue. Histopathologically it was pleomorphic adenoma. There was no reference to pleomorphic adenoma in ectopic lacrimal gland in children by the above search engines and standard textbooks by Henderson's orbital tumors, DM Albert and FA Jackobiec and Char DH.
